# Pediatric renal abscess: a 12-year single-center retrospective analysis

**DOI:** 10.1007/s00467-025-07114-4

**Published:** 2026-03-27

**Authors:** Jian-Qun Guo, Mei-Hao Gao, Yi-Jiao Ma, Shi-Lei Jia, Xiao-Jie Gao, Jun Li

**Affiliations:** 1 Division of Pediatric Nephrology, Shenzhen Children’s Hospital, Shenzhen, Guangdong Province China; 2 Pediatric Department, The Hong Kong University of Shenzhen Hospital, Shenzhen, Guangdong Province China

**Keywords:** Renal abscess, Children, Enhanced CT/MRI, Vesicoureteral reflux

## Abstract

**Background:**

Pediatric renal abscess is an uncommon but serious complication of urinary tract infection, often characterized by nonspecific symptoms and delayed diagnosis.

**Methods:**

We retrospectively analyzed 69 children with radiologically confirmed renal abscesses treated between November 2012 and February 2024. Clinical characteristics, laboratory findings, imaging results, management, and outcomes were evaluated across different age groups.

**Results:**

The median patient age was 24.0 months (IQR 7.3-66.8). There was a marked male predominance in infants (1–24 months) versus a female predominance in children over 60 months, with fever present in 98.6% of cases. Diagnosis was confirmed in all patients by contrast-enhanced CT or MRI, which revealed a mean attenuation difference of 28.8 ± 8.0 HU between the abscess and renal parenchyma; ultrasound sensitivity was 79.7%. Urine cultures were positive in 33.3% of cases, predominantly for Escherichia coli. Notably, vesicoureteral reflux was detected in 52.6% of evaluated patients. While surgical intervention was required in only 7.2% (5/69), these patients had a significantly higher rate of acute kidney injury compared to the conservative group (60% vs. 3.1%; P = 0.0018). The median hospital stay was 12.0 days (IQR 9.0–15.0) and was significantly prolonged in patients with left-sided or bilateral involvement (adjusted P = 0.032 and 0.033, respectively).

**Conclusion:**

Most pediatric renal abscesses can be successfully managed conservatively. Vesicoureteral reflux is common, and acute kidney injury is markedly more frequent among children who require surgical treatment.

**Graphical abstract:**

**Figure:**
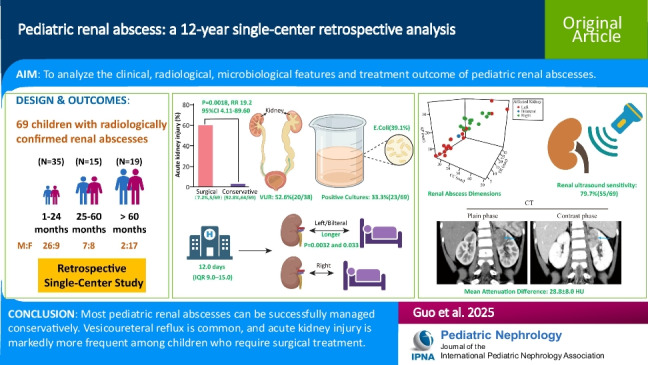
A higher resolution version of the Graphical abstract is available as Supplementary information

**Supplementary Information:**

The online version contains supplementary material available at 10.1007/s00467-025-07114-4.

## Introduction

Renal abscess is an uncommon but serious complication of urinary tract infection (UTI) in children, with potential for permanent renal damage or sepsis if diagnosis is delayed [1, 2]. It typically evolves along a continuum from acute focal bacterial nephritis (AFBN) or refractory febrile UTI (defined as persistent fever beyond 48–72 h despite appropriate antibiotics), progressing to liquefactive necrosis and purulent collection [3, 4]. Advances in contrast-enhanced computed tomography (CT) and magnetic resonance imaging (MRI) have improved detection, yet timely diagnosis remains challenging due to nonspecific symptoms that overlap with uncomplicated UTI [5, 6].

Renal abscesses in children typically result from ascending UTIs, with predisposing factors including vesicoureteral reflux (VUR), obstructive uropathies, and neurogenic bladder, etc. [2, 7]. The true prevalence of VUR in patients with renal abscess remains poorly defined. Bacteremia was historically a key mechanism, often involving Streptococcus species. With widespread antibiotic use and vaccination, the predominant pathogens have shifted to Escherichia coli and other Gram-negative bacteria [8–10].

Despite advances in pediatric renal abscess research, important gaps remain. Most studies have small sample sizes, limiting the reliability and generalizability of their findings. Ultrasound is commonly used for initial screening but lacks sensitivity for small or early lesions. No validated quantitative CT biomarker reliably distinguishes mature abscess from focal pyelonephritis in children, though larger attenuation differences between the lesion and normal parenchyma may suggest pus formation. Contrast-enhanced ultrasound (CEUS) offers a radiation-free option with real-time perfusion imaging [11–14], but its role is not well defined. Data on the indications and outcomes of surgical interventions versus antibiotic therapy alone are limited. These gaps underscore the need for larger, modern studies to improve diagnosis, treatment, and understanding of regional pathogen patterns.

This study investigates the clinical, microbiological, and imaging features of pediatric renal abscesses, with emphasis on age-specific patterns, pathogen distribution, diagnostic accuracy, and treatment outcomes, to address current gaps in this population.

## Methods

### Study design

In this retrospective study, we reviewed 151 children discharged with a diagnosis of renal abscess from Shenzhen Children’s Hospital (Guangdong, China) between November 2012 and February 2024. To ensure diagnostic accuracy, we re-examined all contrast-enhanced CT and MRI scans using strict criteria: (1) well-defined hypodense lesions (0–20 Hounsfield Units (HU)) with rim enhancement and perinephric stranding on contrast-enhanced CT [15, 16]; or (2) T2-hyperintense and T1-hypointense lesions with rim enhancement, restricted diffusion on diffusion-weighted imaging (DWI), and low apparent diffusion coefficient (ADC) values on MRI [17]. Of the 151 cases, 69 met these criteria and were included. The remaining 82 cases were excluded because 81 lacked definitive abscess features on contrast-enhanced CT or MRI and were reclassified as acute pyelonephritis, while one case relied solely on ultrasound without confirmatory advanced imaging.

### Acute kidney injury (AKI) definition and staging

AKI was defined and staged according to the KDIGO criteria [18, 19]. Due to inconsistent urine output records, only serum creatinine (SCr) criteria were applied for AKI detection and staging. The baseline SCr was determined hierarchically from the electronic medical record: (1) the most recent pre-illness SCr when available; (2) the lowest SCr during the hospitalization after hemodynamic stabilization and adequate hydration [20]; (3) the lowest post-discharge follow-up SCr values when available. For patients without any information about baseline SCr, age- and sex-specific reference ranges from Chinese pediatric standards (WS/T 780–2021) were used. KDIGO stages were subsequently assigned based on SCr thresholds.

### CT attenuation measurement

CT attenuation was quantified in HU using values reported in the original radiology reports. The minimum HU within a radiologist-defined region of interest (ROI) in the abscess cavity was recorded. The CT attenuation difference (ΔHU) was calculated as the HU of adjacent normal renal parenchyma minus the HU of the abscess cavity on the same slice. All values were taken directly from the original radiology reports without further adjustment.

### Data collection

Patients were divided into three age groups based on developmental stages: infants and toddlers (1–24 months), preschool children (25–60 months), and school-age children (> 60 months). Data on demographics, clinical features, imaging findings, laboratory results, and treatments outcomes were extracted from electronic medical records. Sonographic resolution was defined as complete disappearance of the hypoechoic abscess lesion (e.g., an anechoic or fluid-filled area). Time to resolution was measured from the start of antibiotics to the ultrasound confirming clearance. Prior antibiotic exposure was defined as any systemic antibiotic given within 48 h before urine collection. Structural urinary tract abnormalities were assessed by renal ultrasound, contrast-enhanced CT/MRI, or voiding cystourethrogram (VCUG).

### Statistical analysis

Continuous variables were summarized as mean ± standard deviation (SD) for normally distributed data and median with interquartile range (IQR) for non-normally distributed data. The Student’s t-test was utilized for pairwise comparisons of normally distributed data, whereas the Mann–Whitney U test was employed for non-normally distributed data. One-way analysis of variance (ANOVA) was utilized for normally distributed data in multiple group comparisons, while the Kruskal–Wallis test was applied for non-normally distributed data. Categorical variables were assessed through the Chi-square test or Fisher’s exact test when expected cell counts were less than 5. The Bonferroni correction was utilized to account for multiple comparisons. A two-tailed P-value of less than 0.05 was deemed statistically significant. Data analysis was conducted utilizing GraphPad Prism version 9.5.0.

## Results

### Clinical characteristics

The study comprised 69 patients diagnosed with renal abscesses, with a median age of 24 months (interquartile range [IQR]: 7.3, 66.8 months) and a male-to-female ratio of 35:34. Age-stratified analysis indicated a male predominance in the 1–24-month age group (26 males compared to 9 females) and female predominance in the > 60-month age group (17 females compared to 2 males). Abscess location was left-sided in 32 patients (46.4%), right-sided in 30 (43.5%), and bilateral in 7 (10.1%).

The most common clinical manifestations include fever, present in 98.6% of patients (68/69), followed by gastrointestinal symptoms (nausea and/or vomiting) in 26.1% (18/69) and abdominal pain in 29.0% (20/69). Less frequent symptoms included loin pain in 5.8% (4/69), abdominal distension in 1.4% (1/69), and respiratory symptoms in 14.5% (10/69). Foul-smelling urine and oliguria were each reported in 1.4% (1/69). Additionally, 20.3% of patients (14/69) had recurrent UTIs, and 5.8% (4/69) had a prior history of fever of unknown origin, as detailed in Table 1.

**Table 1:** Demographic and clinical characteristics of pediatric patients with renal abscesses, stratified by age group

Variables	All patients (*N* = 69)	1–24 months (*N* = 35)	25–60 months (*N* = 15)	> 60 months (*N* = 19)	*P* value
Gender					**< 0.0001**
Male	50.7% (35/69)	74.3% (26/35)	46.7% (7/15)	10.5% (2/19)	
Female	49.3% (34/69)	25.7% (9/35)	53.3% (8/15)	89.5% (17/19)	
Age group distribution	100% (69/69)	50.7% (35/69)	21.7% (15/69)	27.6% (19/69)	
Median age (months)	24.0 (IQR: 7.3–66.8)	7.3 (IQR: 4.0–9.5)	43.4 (IQR: 36.0–53.8)	90.0 (IQR: 78.1–106.8)	**< 0.0001**
Abscess location					**0.942**
Left kidney	46.4% (32/69)	45.7% (16/35)	46.7% (7/15)	47.4% (9/19)	
Right kidney	43.5% (30/69)	42.9% (15/35)	40.0% (6/15)	47.4% (9/19)	
Bilateral	10.1% (7/69)	11.4% (4/35)	13.3% (2/15)	5.2% (1/19)	
Recurrent infection*	26.1% (18/69)	37.1% (13/35)	6.7% (1/15)	21.5% (4/19)	**0.045**
Hospital stays (days)	12.0 (IQR: 9.0–15.0)	12.0 (IQR: 9.0–15.6)	14.0 (IQR: 10.5–15.5)	11.0 (IQR: 9.5–13.0)	**0.3723**
Symptoms					
Fever	98.6% (68/69)	97.1% (34/35)	100.0% (15/15)	100.0% (19/19)	
Abdominal pain	29.0% (20/69)	–^†^	53.3% (8/15)	63.2% (12/19)	
Nausea and vomiting	26.1% (18/69)	11.4% (4/35)	53.3% (8/15)	31.6% (6/19)	
Cough and runny nose	14.5% (10/69)	20.0% (7/35)	6.7% (1/15)	10.5% (2/19)	
Loin pain	5.8% (4/69)	–^†^	6.7% (1/15)	15.8% (3/19)	
Abdominal distension	1.4% (1/69)	–^†^	0.0% (0/15)	5.3% (1/19)	
Dysuria	5.8% (4/69)	2.9% (1/35)	0.0% (0/15)	15.8% (3/19)	
Ultrasound abnormalities					
Enlarged kidney	52.2% (36/69)	48.6% (17/35)	60.0% (9/15)	52.6% (10/19)	**0.75**
Hydronephrosis	29.0% (20/69)	40.0% (14/35)	13.3% (2/15)	21.1% (4/19)	**0.089**
Ureteral dilation	13.0% (9/69)	22.9% (8/35)	0.0% (0/15)	5.3% (1/19)	**0.023**
Duplicated system	4.3% (3/69)	0.0% (0/35)	13.3% (2/15)	5.3% (1/19)	**0.091**
VUR Detection	52.6% (20/38)	62.5% (15/24)	16.7% (1/6)	50.0% (4/8)	**0.042**
Elevated markers					
Blood WBC^‡^	65.2% (45/69)	62.9% (22/35)	66.7% (10/15)	68.4% (13/19)	**0.895**
Blood CRP^‡^	85.5% (59/69)	82.9% (29/35)	93.3% (14/15)	84.2% (16/19)	**0.674**
Urine WBC	78.3% (54/69)	85.7% (30/35)	46.8% (7/15)	89.5% (17/19)	**0.005**
Urine RBC	39.1% (27/69)	31.4% (11/35)	46.8% (7/15)	47.4% (9/19)	**0.379**
Urine LE^§^	59.4% (41/69)	71.4% (25/35)	33.3% (5/15)	57.9% (11/19)	**0.022**
Treatment outcomes					
Surgical intervention ^¶^	7.2% (5/69)	5.7% (2/35)	13.3% (2/15)	5.3% (1/19)	**0.615**
Acute kidney injury ^#^	7.2% (5/69)	11.4% (4/35)	6.7% (1/15)	0.0% (0/19)	**0.246**

^*^ represents cases with recurrent urinary tract infections and recurrent fever of unknown origin

^†^ indicates that patients under 24 months of age are often unable to express feelings of abdominal pain or loin pain or abdominal distension

^‡^ denotes elevated with white blood cell count (WBC > 15 × 10⁹/L) or C-reactive protein (CRP > 30 mg/L)

^§^ represents Leukocyte Esterase (LE), a urinary marker of infection

^¶^ refers to surgical intervention, defined as drainage or surgical procedures for renal abscesses.# indicates acute kidney injury (AKI) defined by the Kidney Disease: Improving Global Outcomes (KDIGO) criteria

### Laboratory findings

Most patients showed elevated inflammatory markers: C-reactive protein in 85.5% (59/69), leukocytosis in 65.2% (45/69), pyuria in 78.3% (54/69), hematuria in 39.1% (27/69), and positive urine leukocyte esterase in 59.4% (41/69). Prior antibiotic exposure was documented in 92.7% (64/69) of patients. Urine cultures were positive in 28.1% (18/64) of the antibiotic-exposed group versus 80.0% (4/5) of the unexposed group (Fisher’s exact test, *P* = 0.0355; OR 9.83, 95% CI 1.07–41.35). Pathogens were isolated from urine in 23/69 patients (33.3%; n = 24 isolates). Escherichia coli predominated (9 isolates,39.1%), followed by Enterococcus spp. (6, 26.1%), Pseudomonas aeruginosa (3, 13.0%), and Enterobacter cloacae (2, 8.7%). One isolate each of Klebsiella oxytoca, Candida glabrata (in one patient with initial sterile culture with prior antibiotics, later positive after prolonged treatment), Corynebacterium glucuronolyticum, and an unspecified Gram-negative bacterium (low count post-antibiotics) was identified. Blood cultures were positive in 3.0% (2/66), growing Klebsiella pneumoniae and Streptococcus constellatus. Purulent fluid from renal abscesses yielded growth in four patients (Table 2).

**Table 2:** Characteristics and outcomes of surgical patients with renal abscess

Patient No	Age (months)	Sex	Affected kidney	Abscess size^a^	VCUG result	Blood culture	Urine culture	Purulent fluid culture	AKI stage^b^	Hospital stays (days)	Duration of pre-operative antibiotic therapy	Time to ultrasound resolution (days)	Indication(s) for surgery
4	7.3	M	Left	NA	Right Grade III, megaureter, neurogenic bladder, ureteral diverticulum	*Klebsiella oxytoca*	*Klebsiella oxytoca*	*Klebsiella oxytoca*	III	44	14	66	Persistent left renal subcapsular/intraparenchymal abscess after 14 days of IV antibiotics with poor spontaneous resolution
10	20.3	M	Left	24 × 19 × 23	Not performed	Negative	Initially negative; later *Candida glabrata*	Not performed	II	42	18	33	Persistent fever and progressive enlargement of the left renal abscess after 18 days of intravenous antibiotics
24	48	F	Bilateral	19 × 17 × 19	Not performed	*Klebsiella pneumoniae*	Negative	*Klebsiella pneumoniae*	I	18	10	≥ 73^c^	Septic shock with increasing abscess size and ongoing fever despite 10 days of intravenous antibiotics
27	25	M	Right	NA	Not performed	*Streptococcus constellatus*	NA	*Streptococcus constellatus*	None	28	40	≥ 73^c^	Persistent fever with hepatic and renal abscesses unresponsive to prolonged broad-spectrum antibiotics
31	75	F	Left	51 × 31 × 31	Not performed	NA	NA	*Escherichia coli*	None	13	41	16	Large size with psoas involvement (extra-renal extension)

^a^ Maximum abscess dimensions (craniocaudal × transverse × anteroposterior, mm)

^b^ Acute kidney injury stage defined by KDIGO serum creatinine criteria

^c^ Lesion persisted on final ultrasound (≥ 73 days); patient lost to follow-up (right-censored)

### Imaging findings

**Fig. 1:**
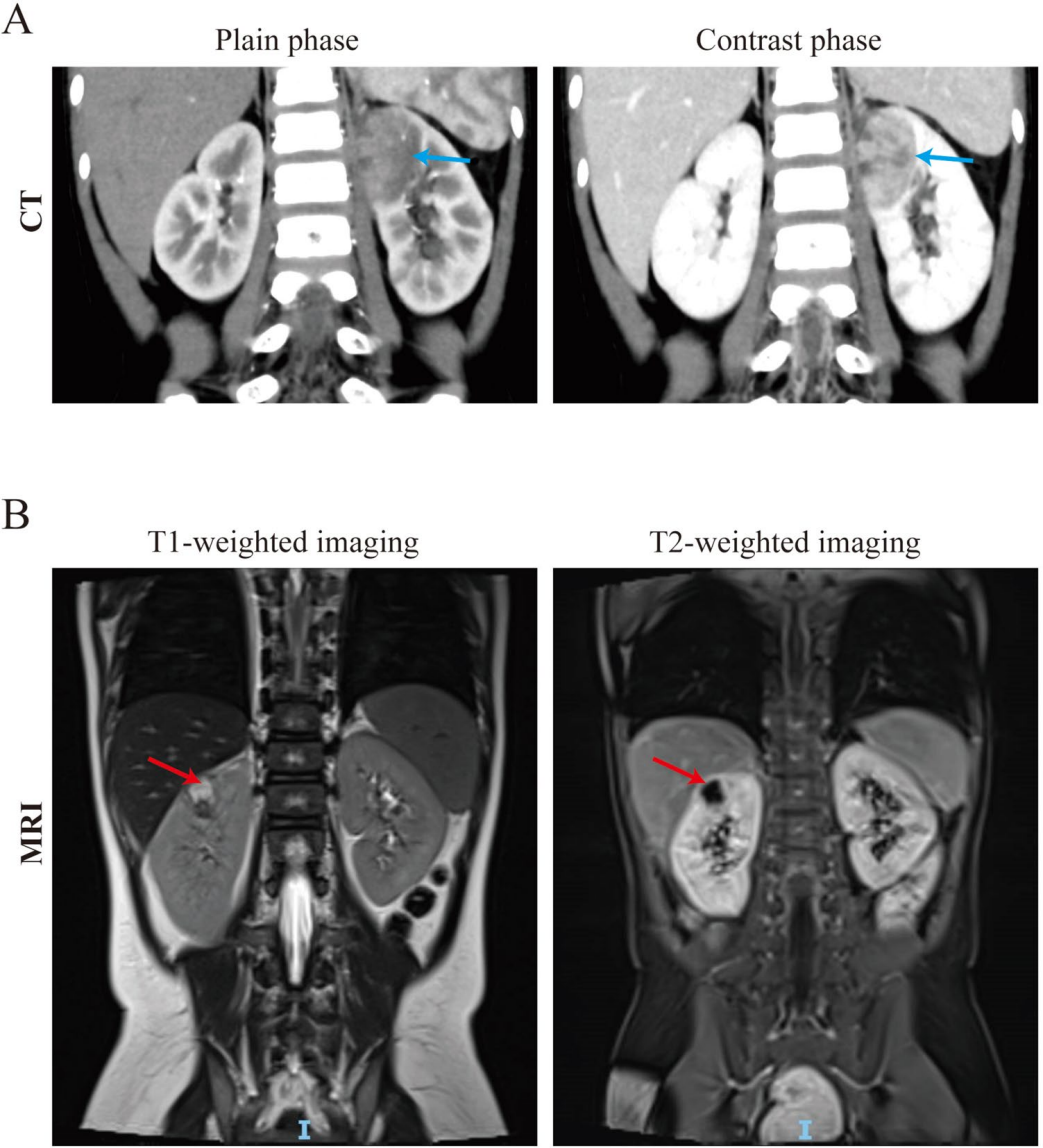
Size of renal abscess and related clinical data.** (A)** Imaging features of renal abscesses on plain and contrast-enhanced CT, with blue arrows marking CT changes. **(B)** MRI images highlighting abscesses with red arrows

**Fig. 2:**
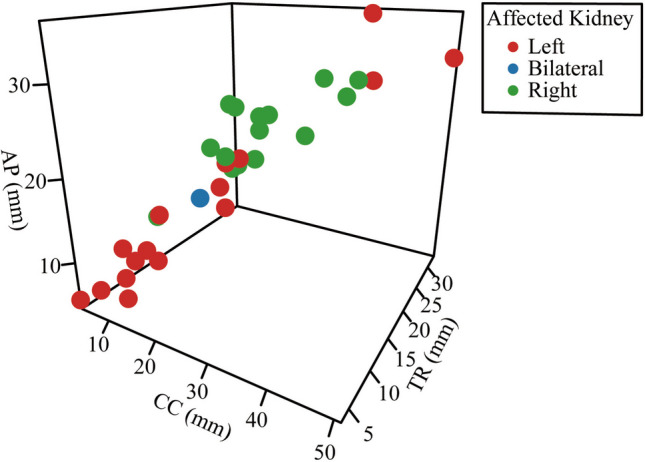
Scatter plot of renal abscess size in three different dimensions: Craniocaudal (CC), Transverse (TR), and Anteroposterior (AP)

Renal ultrasound revealed abnormalities in approximately half of the cases, including enlarged kidneys in 52.2% (36/69), hydronephrosis in 29.0% (20/69), ureteral dilation in 13.0% (9/69), and duplicated collecting systems in 4.3% (3/69) (Table 1). Notably, initial ultrasound failed to detect any of the 14 renal abscesses later confirmed by enhanced CT or MRI, yielding a sensitivity of 79.7% (55/69) (Supplementary Table S1). Enhanced CT was performed in 92.8% (64/69) of patients and MRI in 11.6% (8/69), with three patients undergoing both modalities. Typical imaging features of renal abscess on CT and MRI are shown in Fig. 1A and 1B. Among cases with recorded values, mean abscess attenuation on non-contrast CT was 20.7 ± 7.6 HU (n = 35), with a mean difference of 28.8 ± 8.0 HU from adjacent normal parenchyma on contrast-enhanced CT (n = 37). Renal abscess dimensions are shown in Fig. 2. Abscesses were significantly larger in the left kidney in the craniocaudal (23.9 ± 11.8 mm vs. 12.8 ± 5.6 mm; *P* = 0.0065) and anteroposterior (21.2 ± 8.7 mm vs. 12.6 ± 4.7 mm; *P* = 0.0052) dimensions, but not in the transverse dimension (19.7 ± 8.8 mm vs. 13.7 ± 5.9 mm; *P* = 0.0552). VCUG was declined by 44.9% (31/69) of patients. Among the 38 who underwent VCUG, VUR was present in 52.6% (20/38), with bilateral involvement in 45.0% (9/20). In the subset of 12 culture-positive patients with VCUG, VUR occurred in 60% (3/5) of E. coli cases and 100% (7/7) of non-E. coli cases (*P* = 0.15). Age-stratified VUR rates were 62.5% (15/24) in the 1–24-month group, 16.7% (1/6) in the 25–60-month group, and 50.0% (4/8) in the > 60-month group. Three patients presented complex anomalies: one with bilateral VUR, megaureter, and left ureteropelvic junction obstruction; another with right-sided Grade III VUR, megaureter, ureteral diverticulum, and neurogenic bladder; and a third with left segmental ureteral stenosis confirmed by intravenous pyelogram.

### Treatment outcomes

All 69 patients received empirical antibiotic therapy. Surgical intervention (percutaneous drainage or nephrectomy) was required in 5 patients (7.2%), with a median duration of pre-operative antibiotic therapy of 14 days (IQR 11–18; range 10–40 days). Indications included refractory fever and/or interval abscess enlargement despite appropriate antibiotics (n = 3), abscess rupture with psoas extension (n = 1), and progressive collections following initial deferral of drainage due to extensive necrosis/hyperemia (n = 1) (Table 2).

AKI occurred in 60% (3/5) of surgical vs. 3.1% (2/64) of non-surgical patients (*P* = 0.0018; RR 19.2, 95% CI 4.11–89.60). All resolved within 4 weeks. Median hospital stay was 12.0 days (IQR 9.0–15.0), with no age-group difference (*P* = 0.37). Left-sided or bilateral abscesses had longer hospital stays than right-sided (adjusted *P* = 0.032 and *P* = 0.033). Sonographic resolution was documented in 41 patients at a median of 27 days (IQR 17–45; range 6–139), with no difference by age group (*P* = 0.67) or surgical vs. conservative management (*P* = 0.93).

Twelve patients underwent surgical intervention after abscess resolution. Eleven patients received anti-reflux ureteral reimplantation for vesicoureteral reflux of at least grade III (unilateral or bilateral), except for one patient whose VCUG and anti-reflux surgery were performed at another hospital, for which we have no detailed records. The remaining one patient underwent right ureteroneocystostomy, right ureteral remodeling, and adhesiolysis because of distal right ureteral stricture and right megaureter.

## Discussion

Renal abscess is a rare yet serious inflammatory condition characterized by purulent collections within the renal parenchyma. Historically, diagnosis was challenging due to limited imaging modalities, often resulting in delayed recognition, prolonged hospitalization, or invasive interventions [21]. Advances in antimicrobial therapy and imaging, including contrast-enhanced CT and MRI, have improved detection. Nevertheless, renal abscess remains underrecognized in clinical practice, particularly in pediatric patients [22]. This retrospective, single-center study represents the largest reported cohort of pediatric renal abscesses to date. The objective was to delineate the clinical presentation, diagnostic features, and management strategies in this population.

Fever was observed in nearly all patients within our cohort. Recurrent UTIs were documented in 20.3% (14/69) of cases, and recurrent fever of unknown origin in 5.8% (4/69). These findings are consistent with prior research [7] and underscore a strong association between recurrent UTIs and formation of renal abscesses. Our cohort demonstrated no significant gender difference, which contrasts with previous studies indicating a female predominance [7, 23]. However, age-specific analysis revealed male predominance in the 1–24-month age group and female predominance in children older than 60 months. The findings correspond with gender-specific patterns of UTIs, indicating an ascending route of infection. Renal abscesses were predominantly unilateral. No significant preference for laterality was observed, which contradicts prior reports of right-sided predominance [23–25]. Patients with left-sided or bilateral abscesses had longer hospital stays than those with right-sided involvement. This may reflect greater disease severity or anatomical complexity, as left-sided lesions demonstrated larger craniocaudal and anteroposterior dimensions. These observations suggest that treatment duration and monitoring intensity should be tailored according to abscess location and size, which warrants further investigation.

Urine culture positivity in our cohort was markedly lower than that reported in previous studies [7, 24, 26, 27]. This discrepancy is largely attributable to the exceptionally high rate of antecedent antibiotic exposure, which significantly reduces microbial yield. However, sterile cultures persisted even in one antibiotic-naïve patient, indicating that non-communicating abscesses may not shed organisms into the collecting system. Among positive cultures, gram-negative pathogens predominated, with Escherichia coli accounting for 39.1%, followed by Enterococcus species at 26.1%. Compared with other pediatric cohorts [7, 24], the incidence of Enterococcus species was higher. This finding should be interpreted cautiously, as prior antimicrobial therapy may have selectively eliminated cephalosporin-susceptible organisms.

A striking observation of our study was the low rate of surgical intervention. Several factors likely contributed to this outcome. First, routine renal ultrasound screening in children with febrile UTI enabled early identification of suspected lesions. Second, prompt confirmation with contrast-enhanced CT or MRI facilitated accurate assessment of abscess extent and informed therapeutic decisions. Third, timely initiation of empirical antibiotic therapy likely limited abscess progression and perirenal extension. Finally, accessible pediatric care in China ensured rapid evaluation and management, reducing complications that might require surgery. This combination of early imaging surveillance, rapid confirmation of abscesses, and effective antimicrobial therapy underscores a practical management strategy that can minimize the need for invasive procedures.

A recent study reported an AKI prevalence of 14.6% in children hospitalized for febrile UTI, rising to 30% among those with congenital anomalies of the kidney and urinary tract (CAKUT) [28]. In our cohort, the overall AKI prevalence appeared lower than might be anticipated. Several factors may explain why this rate did not exceed expectations. AKI was defined using serum creatinine-based KDIGO criteria alone, as urine output data were unavailable. Second, many patients had unilateral disease with preserved function in the contralateral kidney, which often prevents detectable rises in serum creatinine despite significant focal inflammation. Third, prompt hydration and antimicrobial therapy likely limited the duration of any serum creatinine elevation. Despite these factors, patients who required surgical intervention had a substantially higher risk of AKI, although the small number of such cases necessitates cautious interpretation.

Ultrasound is a safe initial imaging modality for detecting renal abscess, as it avoids risks associated with impaired renal function or contrast allergy [29]. In our series, renal enlargement on ultrasound was present in 52.2% (36/69) of cases and served as a useful marker of infectious renal involvement. However, ultrasound has lower sensitivity for renal abscess than contrast-enhanced CT or MRI [30]. In many children with renal abscess, ultrasound shows only hypoechoic changes without typical liquefactive necrosis, so reliance on ultrasound alone risks missing diagnoses. The sensitivity of ultrasound in our cohort was 79.7% (55/69), higher than the 33.3% reported previously [23]. This difference may reflect timing of imaging, abscess maturity, size, or internal debris, all of which influence sonographic detection [31]. The application of contrast-enhanced CT or MRI at our center likely improved overall diagnostic yield, though these modalities should be used judiciously due to radiation or contrast concerns.

The prevalence of VUR in our cohort was consistent with a previous report [26], supporting a role for underlying urinary tract anomalies in abscess formation. Nevertheless, this result warrants cautious interpretation, as nearly half of the patients (44.9%) declined VCUG, which inevitably introduces selection bias. VCUG was probably performed more often in children with recurrent or severe infection, which could lead to an overestimation of the true VUR prevalence. Conversely, occult VUR might have been missed, as one study using combined conventional and isotopic cystography detected VUR in 23.6% of additional cases [32]. Thus, the true prevalence in our population could be even higher than expected. Given these considerations, our findings underscore the importance of evaluating VUR in pediatric patients with renal abscess. However, they do not provide sufficient evidence to recommend routine post-infection VCUG for all patients. Instead, VCUG should be selectively indicated for children with atypical clinical features, recurrent UTIs, or radiologic evidence suggestive of anatomical abnormalities. Prospective studies with standardized post infection evaluation, including isotopic cystography, are required to clarify the incidence and clinical significance of VUR in pediatric renal abscess.

All cases in our cohort were confirmed by contrast-enhanced CT or MRI, differing from many prior pediatric series [7, 24, 25]. This approach strengthened diagnostic certainty. To our knowledge, this study is the first to quantify CT attenuation differences (ΔHU) in pediatric renal abscesses. The observed mean ΔHU of 28.8 ± 8.0 HU may serve as an imaging biomarker, but the small sample and incomplete data require cautious interpretation and validation in larger cohorts. CEUS was not used in our series but is increasingly recognized in pediatric nephrology for its lack of ionizing radiation and nephrotoxic contrast, while offering real-time perfusion assessment [33–35]. Future studies that incorporate CEUS could complement CT and MRI to improve noninvasive diagnosis.

Several limitations warrant consideration. The single-center, tertiary-hospital design may introduce selection bias and overestimate disease severity. Although our sample of 69 patients is the largest reported pediatric renal abscess cohort, it remains modest and restricts subgroup analyses. The retrospective design may introduce potential biases and incomplete VCUG evaluation likely underestimates true VUR prevalence. Low culture yield, driven by antibiotic pretreatment in 92.8% of cases, limits microbiologic conclusions. These limitations highlight key directions for future research. Multicenter studies should validate ΔHU thresholds to differentiate abscess from focal bacterial nephritis. Prospective trials integrating CEUS with CT or MRI are needed. Longitudinal cohorts must assess renal outcomes and VUR prevalence using both VCUG and isotopic cystography. Region-specific pathogen surveillance in pretreated children will refine empirical therapy.

## Conclusion

This study describes the largest reported cohort of pediatric renal abscesses confirmed by contrast-enhanced computed tomography or magnetic resonance imaging. Conservative management succeeded in most cases, whereas surgical intervention was associated with markedly higher rates of acute kidney injury. Vesicoureteral reflux was common.

## Supplementary Information

Below is the link to the electronic supplementary material.

Graphical Abstract (PPTX 597 KB)

Supplementary file2 (XLSX 10 KB)

Supplementary file3 (DOCX 16 KB)

## Data Availability

The datasets produced and analyzed in this study are accessible from the corresponding author upon reasonable request.
