# Supplementary material for: Pediatric renal abscess: a 12-year single-center retrospective analysis
Source: Pediatr Nephrol. 2026 Mar 27;41(9):2941–50. doi: 10.1007/s00467-025-07114-4 (PMC13424581; doi:10.1007/s00467-025-07114-4)
Supplement: Supplementary file 1 — Graphical Abstract (PPTX 597 KB) [file 467_2025_7114_MOESM1_ESM.pptx]

## Slide 1
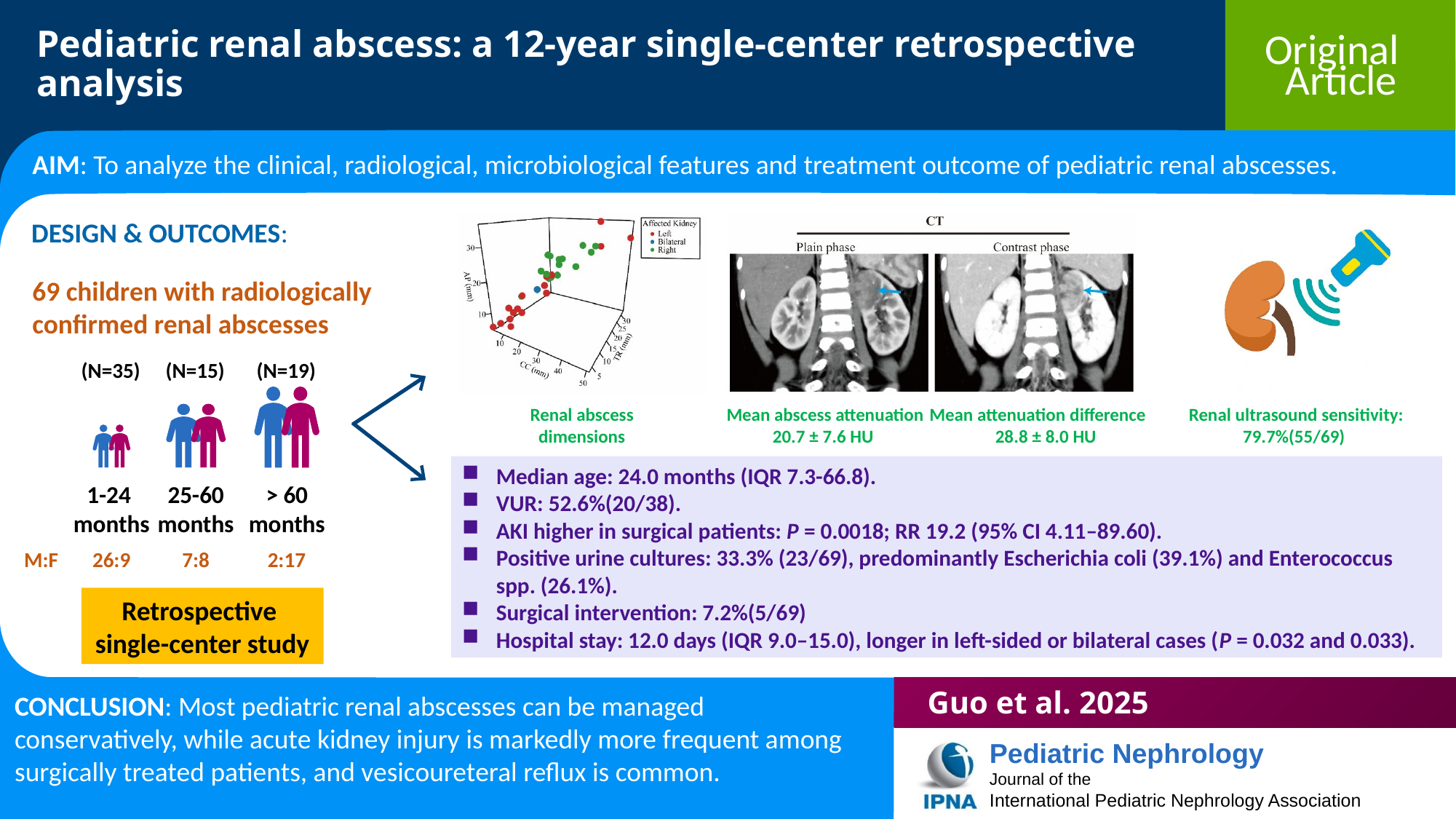

Pediatric renal abscess: a 12-year single-center retrospective analysis
AIM: To analyze the clinical, radiological, microbiological features and treatment outcome of pediatric renal abscesses.
Renal ultrasound sensitivity:
79.7%(55/69)
Renal abscess dimensions
Mean abscess attenuation
20.7 ± 7.6 HU
Mean attenuation difference 28.8 ± 8.0 HU
DESIGN & OUTCOMES:
69 children with radiologically confirmed renal abscesses
(N=35)
(N=15)
(N=19)
Median age: 24.0 months (IQR 7.3-66.8).
VUR: 52.6%(20/38).
AKI higher in surgical patients: P = 0.0018; RR 19.2 (95% CI 4.11–89.60).
Positive urine cultures: 33.3% (23/69), predominantly Escherichia coli (39.1%) and Enterococcus spp. (26.1%).
Surgical intervention: 7.2%(5/69)
Hospital stay: 12.0 days (IQR 9.0–15.0), longer in left-sided or bilateral cases (P = 0.032 and 0.033).
1-24
months
25-60 months
> 60 months
M:F
26:9
7:8
2:17
Retrospective
single-center study
Guo et al. 2025
CONCLUSION: Most pediatric renal abscesses can be managed conservatively, while acute kidney injury is markedly more frequent among surgically treated patients, and vesicoureteral reflux is common.
