# Supplementary material for: Pediatric renal abscess: a 12-year single-center retrospective analysis
Source: Pediatr Nephrol. 2026 Mar 27;41(9):2941–50. doi: 10.1007/s00467-025-07114-4 (PMC13424581; doi:10.1007/s00467-025-07114-4)
Supplement: Supplementary file 3 — Supplementary file3 (DOCX 16 KB) [file 467_2025_7114_MOESM3_ESM.docx]

Supplementary Table S2-Power. Post-hoc power analyses

| **Comparison (endpoint)** | **Test family / Test** | **Tail(s)** | **α** | **Group proportions / means** | **n₁ / n₂** | **Effect size (specify)** | **Achieved power (1–β)** | **Software & settings** |
| --- | --- | --- | --- | --- | --- | --- | --- | --- |
| AKI: Surgery vs Non-surgery | **Exact** family; Fisher’s exact (two independent proportions) | Two | 0.05 | p₁=0.60, p₀=0.03125 | 5 / 64 | Cohen’s *h*=1.788; RR=19.2 | **0.881** | G*Power 3.1.9.7; Proportions: Inequality, two independent groups (Fisher’s exact) |
